# Black esophagus: complete esophageal necrosis with lower esophageal perforation

**DOI:** 10.1055/a-2226-0404

**Published:** 2024-01-23

**Authors:** Lucile Héroin, Pierre-Yves Christmann, François Habersetzer, Pierre Mayer

**Affiliations:** 1 Department of Hepatology and Gastroenterology, Pôle Hépato-digestif, Nouvel Hôpital Civil, Hôpitaux Universitaires de Strasbourg (HUS), Strasbourg, France; 2 Digestive Endoscopy, IHU-Strasbourg (Institut Hospitalo-Universitaire), Strasbourg, France; 3 Inserm U1110, Institute for Viral and Liver Diseases, LabEx HepSYS, University of Strasbourg, Faculty of Medicine, Strasbourg, France

Black esophagus or acute esophageal necrosis is a rare condition, mainly reported as case reports in the literature 1 . Its pathogenesis seems to be multifactorial, with ischemia appearing to be the most common etiology in patients with cardiovascular risk factors and chronic medical conditions 2 . Diagnosis can be suggested by computed tomography (CT) findings but is based on upper gastrointestinal endoscopy. Mortality is high (30%). Treatment is conservative in most cases (75.4%), but endoscopic or surgical interventions can be required (25.6%) 3 .

We report the case of a 72-year-old man, with no medical history other than diabetes, which was managed with biguanides, who was admitted to the emergency room because of urinary tract infection with sepsis. He was rapidly transferred to intensive care because of hemodynamic instability.

Biology showed acute inflammation with leukocytosis and hyperlactatemia, and acute renal failure. Blood cultures were positive for *Corynebacterium confusum* and *Streptococcus sanguinis* . *Aspergillus* antigen was also positive. The CT scan with injection and ingestion showed a circumferential hydroaeric infiltration of the distal esophagus, with a suspected esophageal rupture, a left basal pneumonitis, and bilateral pleural effusion ( Fig. 1 , Video 1 ).

**Fig. 1:**
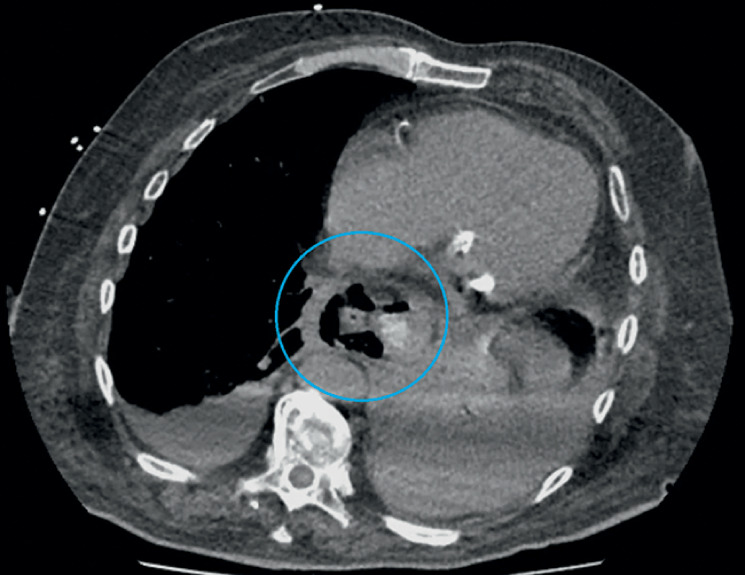
Computed tomography scan with injection and ingestion, showing a circumferential hydroaeric infiltration of the distal esophagus, with a suspected esophageal rupture (blue circle)

Complete esophageal necrosis with lower esophageal perforation.Video 1

Upper gastrointestinal endoscopy was performed and showed complete esophageal necrosis, from the upper esophagus to the gastroesophageal junction ( Fig. 2 ), where the necrosis abruptly stopped. The stomach was unharmed. Opacification showed a perforation in the lower esophagus ( Fig. 3 ).

**Fig. 2:**
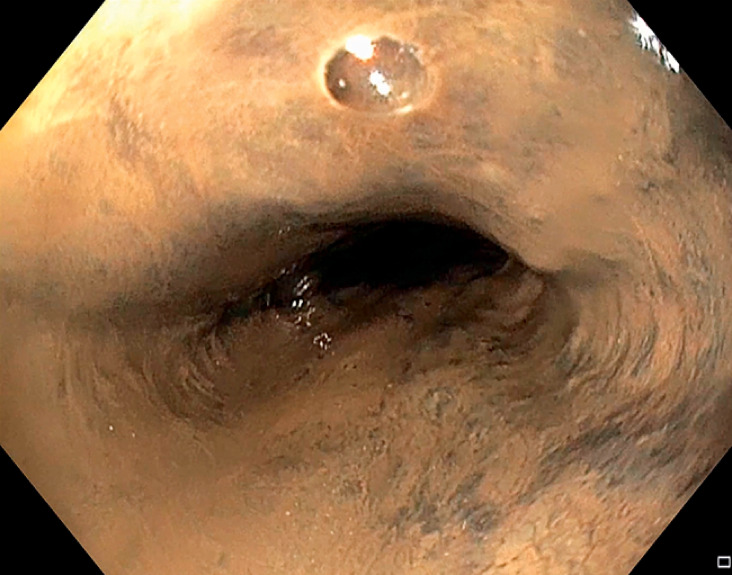
Complete esophageal necrosis.

**Fig. 3:**
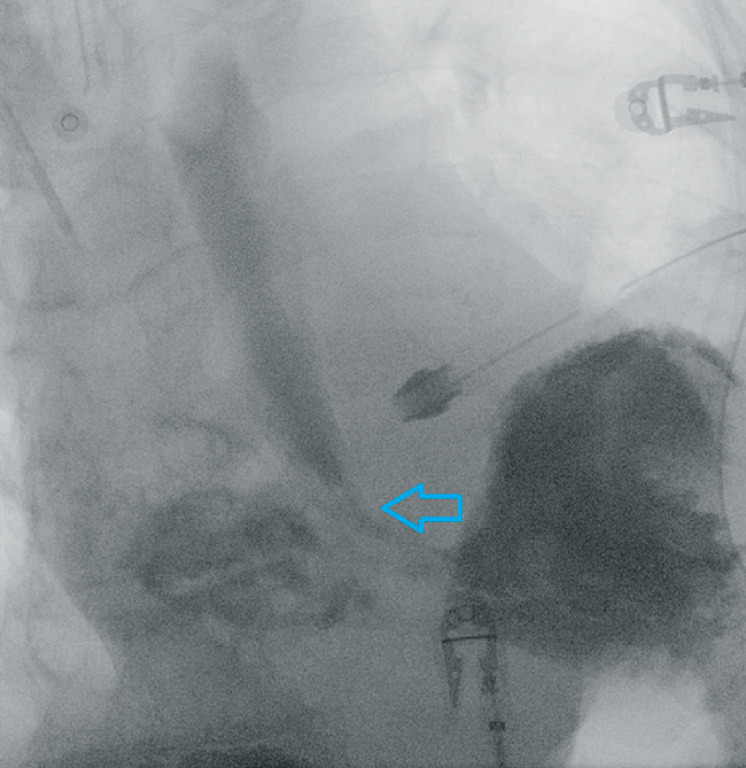
Opacification showing the lower esophageal perforation (arrow).

The patient had expressed his opposition to intensive care when he was alive. The treatment therefore consisted solely of symptomatic therapy, and the patient died within a few hours.

Endoscopy_UCTN_Code_CCL_1AB_2AC_3AH

## Notes

Endoscopy E-Videos https://eref.thieme.de/e-videos
*E-Videos* is an open access online section of the journal *Endoscopy* , reporting on interesting cases and new techniques in gastroenterological endoscopy. All papers include a high-quality video and are published with a Creative Commons CC-BY license. Endoscopy E-Videos qualify for HINARI discounts and waivers and eligibility is automatically checked during the submission process. We grant 100% waivers to articles whose corresponding authors are based in Group A countries and 50% waivers to those who are based in Group B countries as classified by Research4Life (see: https://www.research4life.org/access/eligibility/ ). This section has its own submission website at https://mc.manuscriptcentral.com/e-videos .
